# Genetic Downregulation of Interleukin‐6 Signaling and Arteriolosclerotic Cerebral Small Vessel Disease: A Drug Target Mendelian Randomization Analysis

**DOI:** 10.1161/JAHA.124.041814

**Published:** 2025-11-03

**Authors:** Larissa Ange Tchuisseu‐Kwangoua, Murad Omarov, Alexey Shatunov, Hugh S. Markus, Joseph Kamtchum‐Tatuene, Marios K. Georgakis

**Affiliations:** ^1^ Clinical Trial Service Unit and Epidemiological Studies Unit, Nuffield Department of Population Health University of Oxford Oxford UK; ^2^ HYRICCA (Hypertension et Risque Cardiovasculaire des Camerounais) Research Team, Faculty of Medicine and Biomedical Sciences University of Yaoundé 1 Yaoundé Cameroon; ^3^ Institute for Stroke and Dementia Research, LMU University Hospital LMU Munich Munich Germany; ^4^ Stroke Research Group, Department of Clinical Neurosciences University of Cambridge Oxford UK; ^5^ Wolfson Centre for Prevention of Stroke and Dementia, Nuffield Department of Clinical Neurosciences University of Oxford Oxford UK; ^6^ Program in Medical and Population Genetics and Cardiovascular Disease Initiative Broad Institute of MIT and Harvard Cambridge MA USA

**Keywords:** arteriolosclerosis, cerebral small vessel disease, human genetics, inflammation, interleukin‐6, stroke, Cerebrovascular Disease/Stroke, Precision Medicine, Intracranial Hemorrhage, Cognitive Impairment, Vascular Disease

## Abstract

**Background:**

Arteriolosclerotic cerebral small vessel disease (cSVD) is a leading cause of stroke and dementia, yet no disease‐modifying therapies exist. Anti‐inflammatory strategies targeting IL‐6 (interleukin‐6) signaling have shown efficacy in preventing atherosclerotic cardiovascular disease, but their potential in arteriolosclerotic cSVD remains unexplored. We investigated whether genetically downregulated IL‐6 signaling is associated with clinical, imaging, and pathological manifestations of arteriolosclerotic cSVD.

**Methods:**

We applied 2‐sample Mendelian randomization using (1) 26 genetic variants near *IL6R* (interleukin‐6 receptor) associated with circulating C‐reactive protein levels and (2) rs2228145, a well‐characterized *IL6R* missense variant, as proxies of IL‐6 signaling downregulation. Outcomes included clinical (small vessel stroke, magnetic resonance imaging‐defined lacunar stroke, nonlobar intracerebral hemorrhage, vascular dementia), imaging (white matter hyperintensity volume, extensive basal ganglia perivascular space, nonlobar/mixed cerebral microbleeds), and pathological (arteriolosclerosis burden in autopsy) traits of cSVD, as well as atherosclerosis traits (ultrasound‐defined carotid plaque, large artery stroke) as positive controls. We used inverse‐variance weighting and the Wald ratio estimator for primary analyses. Mendelian randomization‐Egger regression, weighted median, and weighted mode estimators were used as sensitivity analyses.

**Results:**

Genetically downregulated IL‐6 signaling (30% decrement in C‐reactive protein via 26 *IL6R* variants) was not associated with small vessel stroke (odds ratio [OR], 1.02 [95% CI, 0.95–1.10]), magnetic resonance imaging‐confirmed lacunar stroke (OR, 0.95, [95% CI, 0.81–1.11]), nonlobar intracerebral hemorrhage (OR, 1.04 [95% CI, 0.72–1.50]), or vascular dementia (OR, 1.09 [95% CI, 0.95–1.25]). Similarly, we found no significant association with cSVD imaging biomarkers or pathology‐defined arteriolosclerosis. As expected, genetically downregulated IL‐6 signaling was associated with lower odds of large artery stroke (OR, 0.79 [95% CI, 0.74–0.84]) and carotid plaque (OR, 0.88 [95% CI, 0.83–0.94]). Results were consistent across sensitivity analyses and when using the rs2228145 missense variant to proxy IL‐6 signaling downregulation.

**Conclusions:**

Unlike atherosclerotic traits, genetically proxied IL‐6 signaling downregulation is not associated with clinical, imaging, or pathological manifestations of arteriolosclerotic cSVD. These genetic findings suggest that targeting IL‐6 signaling is unlikely to yield effects on cSVD prevention comparable with those expected for atherosclerotic disease.

**Nonstandard Abbreviations and Acronyms**
CASPER: Colchicine After Stroke Event to Prevent Event Recurrence
CHARGE: Cohorts for Heart and Aging Research in Genomic Epidemiology
CMB: cerebral microbleed
CONVINCE: Colchicine for Prevention of Vascular Inflammation in Non‐Cardioembolic Ischemic Stroke
cSVD: cerebral small vessel disease
EPVS: enlarged perivascular space
HRC: Haplotype Reference Consortium
ICH: intracerebral hemorrhage
MEGAVCID: Mega Vascular Cognitive Impairment and Dementia
MR: Mendelian randomization
NINDS‐AIREN: National Institute of Neurological Disorders and Stroke‐Association Internationale pour la Recherche et l'Enseignement en Neurosciences
RIISC‐THETIS: Reducing Inflammation in Ischemic Stroke With Colchicine–Ticagrelor in High‐Risk Patients‐Extended Treatment in Ischemic Stroke
ROSMAP: Religious Orders Study and Memory and Aging Project
TOAST: Trial of Org 10 172 in Acute Stroke Treatment
WMH: white matter hyperintensity

**Clinical Perspective**

**What Is New?**

- Although inhibition of IL‐6 (interleukin‐6) signaling shows promising results in reducing atherosclerotic cardiovascular risk, its potential in preventing arteriolosclerotic cerebral small vessel disease remains unexplored.
- In this drug‐target Mendelian randomization study, no significant associations were found between genetically proxied inhibition of IL‐6 signaling and the risk of clinical, imaging, or pathological manifestations of arteriolosclerotic cerebral small vessel disease.

**What Are the Clinical Implications?**

- Our findings suggest that pharmacological therapies targeting IL‐6 signaling may not be effective in reducing the burden of arteriolosclerotic cerebral small vessel disease, which may have implications for ongoing and planned clinical trials investigating anti‐inflammatory treatments for stroke prevention.

Cerebral small vessel disease (cSVD) encompasses a diverse set of clinical and radiological phenotypes and accounts for about 25% of ischemic strokes and nearly all cases of intracerebral hemorrhage (ICH).^1^, ^2^ It is the leading cause of vascular dementia^3^, ^4^, ^5^ and an independent risk factor for all‐cause and vascular mortality.^6^, ^7^ Magnetic resonance imaging (MRI) markers of cSVD, such as lacunes, white matter hyperintensities (WMHs), enlarged perivascular spaces (EPVS), and cerebral microbleeds (CMBs), are highly prevalent in the aging population, appearing in up to 90% of individuals aged ≥65 years.^8^, ^9^, ^10^ Despite the significant public health burden, the mechanisms underlying cSVD remain largely elusive, which has impeded the development of effective disease‐modifying therapies.^11^, ^12^, ^13^

Anti‐inflammatory treatments targeting the IL (interleukin)‐6 signaling pathway are emerging as preventive strategies for cardiovascular disease.^14^ In phase 3 trials of patients with coronary artery disease, drugs targeting upstream regulators of IL‐6 signaling, such as canakinumab^15^ and colchicine,^16^, ^17^ have shown significant reductions in adverse cardiovascular events including ischemic stroke. However, it remains uncertain whether these benefits extend to nonatherosclerotic vascular pathologies. Arteriolosclerosis of the deep perforating arterioles, the most common pathology finding in cSVD,^18^ is speculated to have an immune component, but the exact inflammatory mediators driving its progression remain undetermined.^19^

This uncertainty complicates patient selection for trials of anti‐inflammatory treatments for secondary ischemic stroke prevention. The CONVINCE (Colchicine for Prevention of Vascular Inflammation in Non‐Cardioembolic Ischemic Stroke) trial evaluated the benefit of colchicine in patients with noncardioembolic ischemic strokes, including those attributed to cSVD.^20^ Although the study did not show a significant reduction in the primary end point of recurrent vascular events in the intention‐to‐treat analysis, a promising reduction was observed in the per‐protocol analysis, excluding patients who were nonadherent to colchicine.^20^ Ongoing trials, such as CASPER (Colchicine After Stroke Event to Prevent Event Recurrence) or RIISC‐THETIS (Reducing Inflammation in Ischemic Stroke With Colchicine and Ticagrelor in High‐Risk Patients‐Extended Treatment in Ischemic Stroke),^21^, ^22^ are also assessing colchicine for secondary prevention in ischemic stroke and include patients with cSVD‐related infarcts.

Human genetics can offer key insights into causal disease mechanisms and inform drug development. Genetically supported drug targets are 2.6 times more likely to result in approved treatments.^23^ Variants near the gene encoding *IL6R* (IL‐6 receptor) are associated with downregulated IL‐6 signaling and have served as proxies to study the potential effect of pharmacological IL‐6 inhibition on various cardiovascular outcomes.^24^, ^25^, ^26^, ^27^, ^28^, ^29^, ^30^, ^31^ Evidence from these genetic studies and findings from clinical^32^ and population‐based studies^33^ have strengthened interest in IL‐6 signaling inhibition as a preventive strategy in atherosclerotic cardiovascular disease, with several phase 3 trials currently underway (ZEUS [A Research Study to Look at How Ziltivekimab Works Compared to Placebo in People With Cardiovascular Disease, Chronic Kidney Disease and Inflammation]‐[NCT05021835], ATHENA [A Research Study Looking Into How Ziltivekimab Works Compared to Placebo in Participants With Heart Failure and Inflammation]‐[NCT06200207], HERMES [Effects of Ziltivekimab Versus Placebo on Morbidity and Mortality in Patients With Heart Failure With Mildly Reduced or Preserved Ejection Fraction and Systemic Inflammation]‐[NCT05636176], ARTEMIS [A Research Study to Look at How Ziltivekimab Works Compared to Placebo in People With a Heart Attack]‐[NCT06118281]).^34^, ^35^, ^36^, ^37^ We performed drug target Mendelian randomization (MR) to explore whether IL‐6 inhibition could also be a viable option for preventing cSVD‐related complications. We assessed whether genetically proxied IL‐6 signaling downregulation is associated with clinical manifestations, imaging biomarkers, and pathological hallmarks of arteriolosclerotic cSVD, using atherosclerosis‐related traits as positive controls.

## METHODS

**Figure 1:**
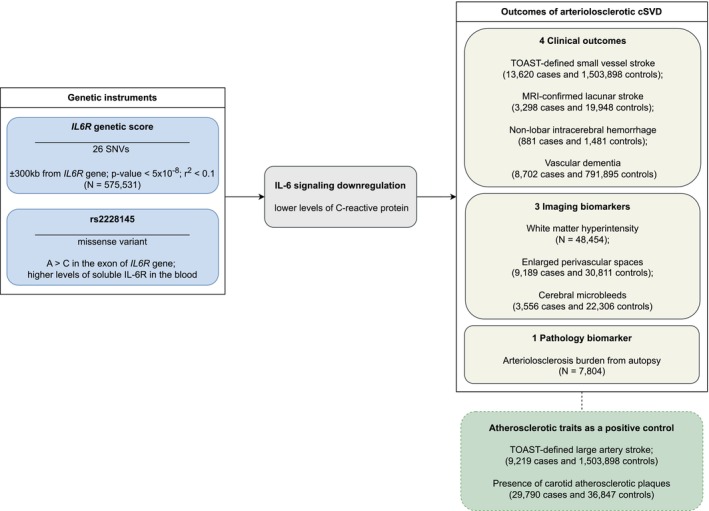
Summary of the study design and of the data sources used for the analysis. cSVD indicates cerebral small vessel disease; IL‐6, interleukin‐6; *IL6R*, interleukin‐6 receptor; MRI, magnetic resonance imaging; SNV, single nucleotide variant; and TOAST, Trial of Org 10 172 in Acute Stroke Treatment.

This report is compliant with the Strengthening the Reporting of Observational Studies in Epidemiology Using Mendelian Randomization statement (checklist presented in Table S1).^38^, ^39^ A graphical overview of the study design is presented in Figure 1. The data sources used for the analyses are provided in Table S2. We applied 2‐sample MR, which uses selected genetic instruments to assess the relationship between genetically proxied IL‐6 signaling and various cSVD manifestations.

### Genetic Instruments

We used 2 sets of genetic instruments to proxy IL‐6 signaling downregulation. First, according to an approach we previously developed,^26^, ^27^, ^29^, ^31^ a list of 26 genetic variants were selected based on (1) their location within a window including the *IL6R* gene ±300 kb, (2) their association with circulating C‐reactive protein (CRP) levels at a *P* value <5×10^−8^ in a genome‐wide association studies (GWAS) meta‐analysis of 575 531 individuals of European ancestry from the CHARGE (Cohorts for Heart and Aging Research in Genomic Epidemiology) consortium and the UK Biobank,^40^ and (3) their lack of strong correlation between them due to linkage disequilibrium (clumped at *r* ^2^ <0.1). Second, we used the missense single nucleotide polymorphism (SNP) rs2228145, which is part of the 26‐variant instrument and has strong evidence for a functional effect leading to downregulation of IL‐6 signaling and a significant reduction in circulating levels of CRP.^24^ This SNP is located within the *IL6R* gene and leads to a change from adenine (A) to cytosine (C) at position 1073 in the 9th exon of the gene resulting in a Asp358 → Ala358 change in the IL‐6R (interleukin‐6 receptor) protein. The Asp358Ala substitution leads to an increase in the shedding of the membrane‐bound IL‐6R, thus decreasing the availability of membrane‐bound IL‐6R and increasing the production of circulating soluble IL‐6R.^41^, ^42^

For both instruments, we calculated the *F* statistics of the individual variants, as well as the cumulative variance of CRP explained by all variants in an additive model. All instruments had *F* statistics >10, indicating sufficient instrument strength. Detailed information on the instruments is provided in Table S3.

### Analysis of Individual‐Level UK Biobank Data

We used data from the UK Biobank to explore the effects of both instruments on circulating CRP levels. The UK Biobank is a UK‐wide prospective cohort of 503 317 adults aged 39 to 73 years, recruited between 2006 and 2010. The design and rationale of the study have been described previously.^43^ Eligible participants were registered with the National Health Service in England, Scotland, or Wales, and lived close to 1 of the 22 assessment centers in these regions. Details on demographics, lifestyle, and medical history were collected during the visit to the assessment centers. Anthropometric data and biological (blood, urine, and saliva) samples were also collected by trained personnel. Participants gave their written informed consent to be followed up through a national record linkage.

The UK Biobank contains genotype data for 488 377 participants, with 49 979 genotyped using the UK Biobank Lung Exome Variant Evaluation (UK BiLEVE) array and 438 398 using the UK Biobank Axiom array. Following standard quality control procedures, 968 samples identified as outliers in heterozygosity levels or missing genotype rates were excluded, leading to a total of 487 409 individuals.^44^ Details on preimputation quality checks and imputation processes are provided elsewhere.^44^ Briefly, multiallelic SNPs and those with a minor allele frequency ≤1% were filtered out. Phasing of genotype data was performed using the Segmented HAPlotype Estimation and Imputation Tool version 3 (SHAPEIT3) algorithm.^45^ Genotype imputation was conducted using the IMPUTE4 algorithm^46^ with 2 reference panels: United Kingdom 10,000 Genomes Project (UK10K) haplotype and HRC (Haplotype Reference Consortium). Excluding 519 participants who withdrew from the study (defined by UK Biobank field 190) and 22 626 individuals with missing CRP levels resulted in a total set of 464 264 UK Biobank participants available for the analysis.

For the first instrument, we used the clumping and thresholding method to construct a genetic risk score based on the 26 *IL6R* CRP‐increasing genetic variants and explored differences in CRP between the top and bottom percentiles of the distribution.^47^ The score was calculated with PLINK version 2.00a.^48^ For the rs2228145, we calculated differences in CRP between carriers of the A and C alleles of the variant. We explored the effects of the 2 genetic instruments on ln‐transformed CRP levels in linear regression models adjusted for age as a continuous variable, sex as a categorical variable, and the first 10 principal components of the population structure. We also ran univariable linear regression analyses to calculate the proportion of CRP explained by the 2 instruments. We scaled both instruments to correspond to a reduction in CRP levels that reflects the natural variation observed across carriers of the genetic variants (30% reduction in median CRP levels for individuals in the bottom versus top percentile of the distribution of the 26‐variant genetic score and 18% reduction in median CRP levels for CC versus AA allele carriers).

### Outcomes

We used clinical outcomes of cSVD, namely lacunar stroke, nonlobar ICH, and vascular dementia. In addition, we analyzed associations with MRI markers of cSVD. This allows accurate phenotyping of the features of cSVD by providing information on the more chronic features of cSVD, rather than risk factors for acute infarction. We also studied associations with pathology‐confirmed arteriolosclerosis. We used publicly available summary statistics from GWAS to extract the effects of the selected genetic variants on the outcomes of interest (Table S2).

#### Clinical Manifestations Attributed to Arteriolosclerotic Cerebral Small Vessel Disease

1. Small vessel stroke defined as a clinical syndrome of lacunar stroke without evidence of alternative causes in diagnostic workup, and no evidence of a new cortical or large subcortical (≥15 mm) infarct on computed tomography (CT) or MRI as per TOAST (Trial of Org 10 172 in Acute Stroke Treatment) criteria.^49^

We used data from the cross‐ancestry GWAS meta‐analysis of the GIGASTROKE study (13 620 cases and 1 503 898 controls; 67% European, 25% East Asian, 4% African, 3% South Asian, 1% Hispanic) for our primary analyses.^50^ We also used GIGASTROKE European ancestry subsample with 6811 cases and 1 234 808 controls and data from the MEGASTROKE GWAS study on small vessel stroke with 5386 European cases and 343 560 European controls for sensitivity analyses.^51^

2. MRI‐confirmed lacunar stroke.

Small vessel stroke in the GIGASTROKE database has been subtyped based largely on a diagnosis of a clinical lacunar syndrome combined with CT brain imaging. However, CT imaging may not show lacunar infarcts, particularly in the first 24 hours. MRI provides a more accurate diagnosis of lacunar infarction, because as many as 50% of all patients classified as small vessel stroke based on CT imaging do not have cSVD on MRI.^52^ Therefore, we also studied a population with MRI‐confirmed lacunar stroke defined as a clinical lacunar stroke syndrome with an anatomically corresponding lacunar infarct of ≤15 mm in diameter. We used data from a study of 3298 cases and 19 948 controls (only European ancestry), which included data from a published study (2612 cases)^53^ and 686 newly genotyped samples from the UK DNA Lacunar Stroke 1 and 2.

3. Primary spontaneous ICH originating in nonlobar brain locations (thalamus, internal capsule, basal ganglia, deep periventricular white matter, cerebellum, or brain stem) as confirmed with neuroimaging.

We used data from a GWAS meta‐analysis of 6 case‐control studies of European ancestry only.^54^ Only nonlobar ICH cases (corresponding to 881 cases and 1481 controls) were analyzed, because they are more likely to be causatively related to arteriolosclerosis, as opposed to lobar ICH cases that are more commonly linked to cerebral amyloid angiopathy.^55^

4. Vascular dementia defined according to clinical criteria (*Diagnostic and Statistical Manual of Mental Disorders III to V*, *International Classification of Diseases, Ninth or Tenth Revision* [*ICD‐9* or *ICD‐10*], National Institute of Neurological Disorders and Stroke‐Association Internationale pour la Recherche et l'Enseignement en Neurosciences, and Alzheimer's Disease Diagnostic and Treatment Center).

We used data from a GWAS meta‐analysis of 21 cohorts and consortia performed by the MEGAVCID (Mega Vascular Cognitive Impairment and Dementia) consortium (8702 cases and 753 695 controls; 97% European, 1% African, 1% East Asian, <1% Hispanic).^56^ Only the European‐specific GWAS was used in the analysis made of 3892 cases and 466 606 controls. cSVD is considered 1 of the major contributors to vascular dementia.^3^, ^4^, ^5^

#### Imaging Biomarkers of Arteriolosclerotic Cerebral Small Vessel Disease

1. WMH volume on brain MRI images (T2‐weighted or fluid‐attenuated inversion recovery sequences) quantified with fully automated or semiautomated software.

We extracted data from a GWAS meta‐analysis of predominantly European ancestry participants of the UK Biobank and 23 CHARGE cohorts (N=48 454, 95% European and 5% Black).^57^ WMH volume was inverse‐normal transformed across the cohorts, and association estimates may be interpreted in standard deviation units of WMH volume.

2. Enlarged perivascular spaces (EPVS) defined as fluid‐filled spaces with a signal identical to that of cerebrospinal fluid with a maximum diameter <3 mm, no hyperintense rim on T2‐weighted or fluid‐attenuated inversion recovery sequences, and located in areas supplied by perforating arteries.

We obtained data from a GWAS meta‐analysis of 18 population‐based studies (UK Biobank, cohorts of the CHARGE consortium and the Brrain Imaging, Cognitive, Dementia, and Next‐Generation Genomics [BRIDGET] initiative) including 40 095 stroke‐free participants (97% European, 2% Hispanic, <1% East Asian, <1% Black).^58^ Across the provided locations (white matter, basal ganglia, hippocampus), we focused on EPVS in basal ganglia, because these lesions are more likely to be related to arteriolosclerosis.^59^ Because different scales were used to quantify EPVS across studies, the trait was analyzed as a binary variable with logistic regression (extensive versus nonextensive EPVS; 9189 cases and 30 811 controls) with a cohort‐specific threshold closest to the top quartile (75th percentile) of the semiquantitative scale distribution within each cohort.^58^ Therefore, cases with EPVS burden above this cohort‐specific threshold were classified as extensive EPVS, whereas those below the threshold were classified as nonextensive EPVS.

3. CMBs recognized as small hypointense lesions on susceptibility‐weighted imaging sequences or, to a lesser extent, on T2‐weighted gradient echo sequences.

Data on CMB were extracted from a GWAS meta‐analysis undertaken in population‐based cohorts of the CHARGE consortium and the UK Biobank, totaling 25 862 individuals of all ancestries (3556 cases with microbleeds, of which 2179 were strictly lobar and 1293 deep, infratentorial, or mixed, and 22 306 controls; 97% European, 2% Black, <1% Malay, <1% Chinese).^60^ We focused our analyses on deep or infratentorial CMBs and mixed CMBs, because they are more likely to be causatively related to arteriolosclerosis, as opposed to strictly lobar CMBs that are typically linked to cerebral amyloid angiopathy.^55^

#### Pathology Burden of Arteriolosclerosis

To explore associations with pathology‐confirmed arteriolosclerosis, we extracted data from a GWAS meta‐analysis of data from the US National Alzheimer's Coordinating Center neuropathology study, the ROSMAP (Religious Orders Study and Memory and Aging Project) study, and the ACT (Adult Changes in Thought) study totaling 7804 autopsied participants (all European ancestry).^61^ Arteriolosclerosis burden was analyzed as an ordinal variable ranging from 0 to 3 (0=none, 1=mild, 2=moderate, 3=severe) according to the qualitative assessment of a neuropathologist.^62^

#### Atherosclerosis‐Related Traits as Positive Controls

The purpose of using positive controls was to validate the genetic instruments by assessing their relationship with a well‐established and biologically plausible outcome. Given that several studies have previously reported associations of genetic proxies of IL‐6 signaling downregulation with atherosclerotic cardiovascular disease,^24^, ^25^, ^26^, ^27^, ^28^, ^29^, ^30^, ^31^ atherosclerosis‐related traits were selected as positive controls, thus providing a reference point to contextualize our findings and verify that the genetic instruments function as expected. The following cerebrovascular atherosclerosis traits were used as positive controls:

1. TOAST‐defined large artery atherosclerotic stroke defined as an ischemic stroke with evidence of a ≥50% stenosis in a supplying artery.

We used data from the transancestry GIGASTROKE study (9219 cases and 1 503 898 controls; 67% European, 25% East Asian, 4% African, 3% South Asian, 1% Hispanic).^50^ A European subsample from the same study was used for sensitivity analysis (6399 cases and 1 234 808 controls).

2. Presence of carotid atherosclerotic plaques on carotid ultrasound defined as focal wall structure protruding into the arterial lumen of the distal common or proximal internal carotid artery by ≥0.5 mm or ≥50% of the surrounding intima‐media thickness or with an overall thickness of >1.5 mm as measured from the media‐adventitia interface to the intima‐lumen interface.^63^

We used data from a GWAS meta‐analysis of the UK Biobank and the CHARGE cohorts (29 790 cases and 36 847 controls; all European ancestry).^64^

### Statistical Analysis

We performed 2‐sample MR to explore the associations between genetically proxied downregulation of IL‐6 signaling and the cSVD‐related outcomes. MR analyses were performed using the TwoSampleMR package in R Studio version 3.5.1. For the 26‐variant instrument, we used fixed‐effects inverse variance‐weighted MR as our primary analytical approach, because it has the highest power in absence of pleiotropy.^65^ The Cochran *Q* test was used to assess heterogeneity between the variants as a metric of pleiotropy (statistical significance set at *P*<0.05).^66^ To mitigate potential bias of the inverse variance‐weighted approach due to possible presence of directional horizontal pleiotropy, 3 other MR sensitivity methods were used: MR‐Egger, weighted mode, and weighted median. MR‐Egger regression assumes that genetic instruments are uncorrelated with any pleiotropic effect of the instrument on the outcome.^67^ The intercept of MR‐Egger regression was used as a measure of unbalanced pleiotropy (*P*<0.05 indicated significance). The weighted mode, resistant to outliers, assumes that the most common effect across instrumental variables is consistent with the true causal effect even if the majority of instruments are invalid.^68^ The weighted median assumes that at least 50% of the weight comes from valid SNPs. A leave‐1‐out sensitivity analysis was conducted to evaluate whether individual SNPs could drive the results. For the rs2228145 variant, we applied the Wald ratio method.^69^

The derived odds ratios (ORs) correspond to a 30% or 18% decrement in CRP levels for the 26‐variant instrument and the rs2228145 variant, respectively. All *P* values are 2‐sided. Because we analyzed 8 main outcomes (4 clinical, 3 radiological, 1 pathological), our significance threshold was set at *P*<0.00625 to account for multiple testing using the Bonferroni approach. A *P*<0.05 indicates nominal significance. For clinical outcomes, we performed power calculations estimating the OR range we were sufficiently powered (1−β >0.8) to detect at a nominal significance level (α=0.05).

### Ethics

The UK Biobank obtained approval from the Northwest Multi‐Center Research Ethics Committee (Research Ethics Committee reference for UK Biobank [11/NW/0382]). All participants provided written informed consent. Data for these analyses were accessed under application number 151281. The DNA Lacunar Stroke 2 was approved by East of England Research Ethics Committee (16/EE/0201), and all participants provided signed informed consent. The publicly available GWAS summary statistics were generated by studies that had obtained ethical approval and participant consent for analyses and distribution of summary‐level data, as described in the original publications.

### Data Availability

The genetic variants used as instruments in this analysis along with their weights are provided in Table S3. The GWAS summary data sets for CRP used to weigh the instruments are downloadable from the GWAS catalog under accession number GCST90029070. Summary statistics for GIGASTROKE transancestry analysis of small vessel stroke and large artery stroke are available from the GWAS catalog under accession numbers GCST90104537 and GCST90104538, respectively. Their corresponding European summary statistics were downloaded under the accession number GCST90104543 and GCST90104542. For MEGASTROKE, GWAS summary statistics were obtained from the website https://www.megastroke.org/. Data on extended basal ganglia perivascular space GWAS data were taken from the GWAS catalog number GCST90244154. Additionally, GWAS summary data from the CHARGE consortium on WMH and nonlobar ICH can be obtained from the Database of Genotypes and Phenotypes under accession numbers phs002227.v1.p1 and phs000416.v1.p1, respectively. GWAS summary statistics for CMBs are available through the Data Set Inspector Cerebrovascular Disease Knowledge Portal. GWAS summary statistics for ultrasound‐defined carotid plaque are available from the GWAS catalog under the accession number GCST90454353. GWAS summary statistics for vascular dementia, MRI‐confirmed lacunar stroke, and pathology‐defined arteriolosclerosis burden were provided upon request to the corresponding authors of the articles describing the data sets. UK Biobank data are available upon submission of an application to the UK Biobank.

## RESULTS

### Genetic Instruments for IL‐6 Signaling Downregulation

A total of 464 264 participants from the UK Biobank (54.2% women, median age 58 years) had available genetic data and were included in this analysis (Table 1). A genetic score consisting of the 26 variants included in our main genetic instrument was strongly associated with CRP, explaining 0.45% of its variance in a linear regression model. Individuals in the top percentile of the distribution of the score had 30.3% higher CRP levels on average, when compared with individuals in the bottom percentile (1.48 versus 1.08 mg/dL; Table 2). Among the study participants, there were 220 103 (47.4%) heterozygote and 76 146 (16.4%) homozygote carriers of the C allele of the rs2228145 variant. When compared with homozygote carriers of the A allele, the median serum CRP levels were 9.0% and 19.4% lower in participants with AC and CC genotypes, respectively (median CRP levels: 1.44, 1.31, 1.16 mg/dL for AA, AC, and CC genotypes, respectively; Table 2).

**Table 1:** Baseline Characteristics of the UK Biobank Participants With Available Genetic Data (N=464 264)

Baseline characteristics	Value
Women, n (%)	251 629 (54.2)
Age, mean±SD	57.1±8.1
Smoking status, n (%)	
Never	252 837 (54.46)
Previous	160 380 (34.55)
Current	48 695 (10.49)
CRP levels, mg/L, median (IQR)	1.33 (0.66–2.76)
SBP, mm Hg, mean±SD	137.90±18.60
DBP, mm Hg, mean±SD	82.25±10.12
HbA1c, mmol/mol, mean±SD	36.12±6.75
History of diabetes, n (%)	24 132 (5.20)
History of hypertension, n (%)	138 900 (29.92)
Race and ethnicity, n (%)	
White	437 781 (94.30)
Asian	8907 (1.92)
Black	7157 (1.54)
Mixed	2716 (0.59)

Missing data for smoking (n=2352), SBP (n=490), DBP (n=488), HbA1c (n=21 417), race and ethnicity (n=7703). CRP indicates C‐reactive protein; DBP, diastolic blood pressure; HbA1c, hemoglobin A1c; IQR, interquartile range; and SBP, systolic blood pressure.

**Table 2:** CRP Levels Across the Distribution of the Genetic Instruments for Interleukin‐6 Signaling Downregulation in the UK Biobank (N=464 264)

rs2228145	No.	Median CRP, mg/L	Decrease in median, %	CRP ≥2 mg/L, %
AA	168 015	1.48 [0.73 to 3.02]	Reference	38.7
AC	220 103	1.35 [0.67 to 2.79]	−8.8*	35.7
CC	76 146	1.21 [0.59 to 2.48]	−18.2*	31.4

26‐Variant *IL6R* GRS	No.	Median CRP, mg/L	Decrease in median, % [95% CI]	% CRP ≥2 mg/L
Top 25%	116 071	1.46 [0.71 to 2.98]	Reference	38.2
Bottom 25%	116 091	1.19 [0.59 to 2.49]	−18.5 (−19.2 to −17.2)*	31.6
Top 10%	46 428	1.47 [0.72 to 3.02]	Reference	38.7
Bottom 10%	46 437	1.14 [0.57 to 2.38]	−22.4 (−24.2 to −21.2)*	30.2
Top 5%	23 291	1.48 [0.73 to 3.02]	Reference	38.8
Bottom 5%	23 215	1.11 [0.56 to 2.30]	−25.0 (−27.3 to −23.3)*	29.1
Top 1%	4644	1.48 [0.74 to 3.01]	Reference	37.9
Bottom 1%	4643	1.08 [0.55 to 2.23]	−30.3 (−26.5 to −34.4)*	28.5

Median values are accompanied by the 25th and 75th percentiles [interquartile range]. 95% CIs for the percent decrease in median CRP values were estimated using bootstrap resampling with 10 000 iterations. CRP indicates C‐reactive protein; GRS, genetic risk score; and *IL6R*, gene encoding the interleukin‐6 receptor.

* All results were significant at *P*<2.2×10^−16^ according to pairwise comparisons performed using Dunn's test after Kruskal‐Wallis.

### Genetically Downregulated IL‐6 Signaling and Clinical Arteriolosclerotic cSVD Outcomes

**Figure 2:**
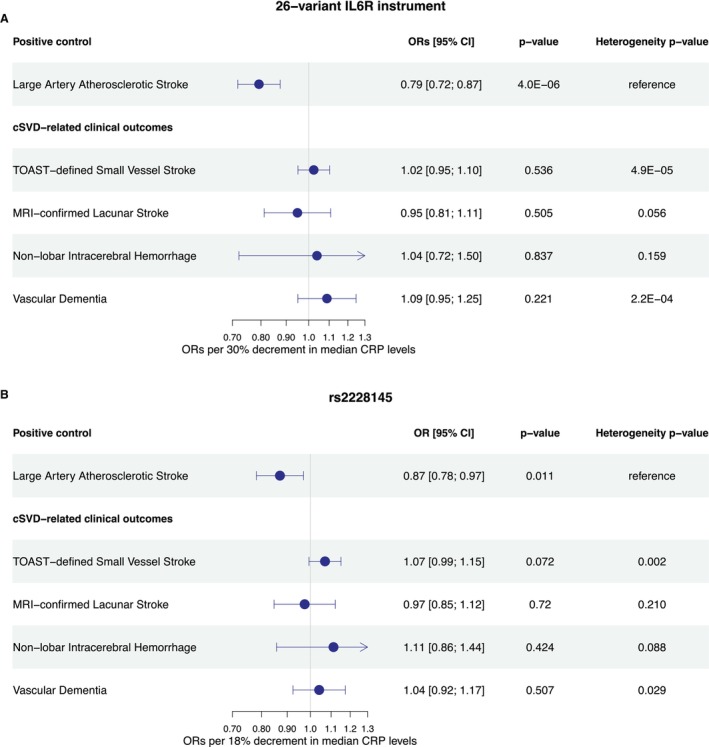
Genetically proxied IL‐6 signaling downregulation and arteriolosclerotic cSVD‐ related clinical outcomes. Results derived from (**A**) a fixed‐effects inverse‐variance weighted Mendelian randomization analysis for a genetic instrument composed of 26 CRP‐lowering variants in the *IL6R* locus and (**B**) the Wald ratio method for the genetic instrument composed of the single rs2228145 variant. Heterogeneity *P* values calculated using the Cochran *Q* statistic represent comparisons between the OR for each outcome and OR for large artery atherosclerotic stroke (positive control). CRP indicates C‐reactive protein; cSVD, cerebral small vessel disease; IL‐6, interleukin‐6; *IL6R*, interleukin‐6 receptor; MRI, magnetic resonance imaging; OR odds ratio; and TOAST, Trial of Org 10 172 in Acute Stroke Treatment.

The *F* statistics for the 26 selected variants ranged from 31 to 2033, thus indicating that the instruments are strong, because *F* statistics >10 typically indicate that the level of weak instrument bias is likely to be small.^70^ In line with previous studies,^29^, ^30^, ^64^, ^71^ genetically downregulated IL‐6 signaling proxied by the 26‐variant genetic instrument was associated with lower odds of large artery atherosclerotic stroke (OR per 30% decrement in CRP levels, 0.79 [95% CI, 0.72–0.87]; *P*=4×10^−6^) in the GIGASTROKE study (Figure 2A). However, we found no significant evidence that genetically proxied downregulation of IL‐6 signaling is associated with cSVD‐related clinical outcomes including TOAST‐defined small vessel stroke (OR, 1.02 [95% CI, 0.95–1.10]; *P*=0.54), MRI‐confirmed lacunar stroke (OR, 0.95 [95% CI, 0.81–1.11]; *P*=0.50), nonlobar intracerebral hemorrhage (OR, 1.04 [95% CI, 0.72–1.50]; *P*=0.84), or vascular dementia (OR, 1.09 [95% CI, 0.95–1.25]; *P*=0.221, Figure 2A). Post hoc power calculations for the 26‐variant instrument suggested that the data sets used for TOAST‐defined small vessel stroke, MRI‐confirmed lacunar stroke, and vascular dementia offered sufficient statistical power (>80%) at a 5% type I error rate to detect associations similar to the one observed for large artery stroke. Specifically, we were sufficiently powered to detect ORs per 30% decrement in CRP of ≤0.90, ≤0.87, and ≤0.78, respectively (Table S4).^72^ Therefore, although the true effect might be different from 0, the observed effect sizes and confidence intervals suggest that any impact of IL‐6 signaling downregulation on cSVD outcomes would be smaller than the effects observed for atherosclerosis‐related end points. For all 4 outcomes, the ORs were significantly higher than the OR for large artery stroke, as indicated by significant heterogeneity estimates (all *P*<0.05; Figure 2A). This suggests that the effect of genetically predicted IL‐6 downregulation varies between atherosclerosis‐driven and cSVD‐related stroke subtypes. With the exception of vascular dementia (Cochran *Q*‐derived *P* value for heterogeneity=0.007 from the inverse variance weighted MR), there was no significant evidence of between‐variant heterogeneity for any of the outcomes. No indication of directional pleiotropy, as assessed by the MR‐Egger regression (with *P*<0.05 considered significant), was detected for any of the outcomes. The alternative MR methods (MR‐Egger regression, weighted mode, and weighted median estimator) for the 26‐variant instrument showed similar effects to that of the inverse variance weighted analysis (Table S5). In addition, the leave‐1‐out sensitivity analyses for clinical outcomes demonstrated that no single SNP disproportionately influenced the results, further supporting the robustness of our findings (Figure S1).

The analysis for the missense variant rs2228145 revealed similar associations, albeit as expected, of wider confidence intervals due to lower statistical power (Figure 2B). Specifically, there was a nominal association between genetically proxied IL‐6 signaling downregulation and lower odds of large artery stroke (OR per 18% decrement in CRP levels, 0.87 [95% CI, 0.78–0.97]; *P*=0.01), but no significant evidence of association with TOAST‐defined small vessel stroke, MRI‐confirmed lacunar stroke, nonlobar intracerebral hemorrhage, or vascular dementia. Sensitivity analyses restricted to individuals of European ancestry for small vessel and large artery stroke showed that results were consistent with those derived from the cross‐ancestry analyses (Table S5).

Previous MR analyses have shown links between genetically proxied inhibition of IL‐6 and a lower risk of small vessel stroke.^29^ To investigate potential sources of discrepancy, such as variations in genetic instruments, population admixture, or outcome definitions, we examined associations using small vessel stroke outcomes from previous GWAS with varying sample sizes and definitions. We found that genetically downregulated IL‐6 signaling was associated with small vessel stroke in the earlier GWAS from MEGASTROKE (OR, 0.85 [95% CI, 0.77–0.95]; *P*=0.003), but this association was attenuated in the larger GIGASTROKE data set (OR, 0.93 [95% CI, 0.82–1.06]; *P*=0.28), which is more enriched with MRI‐confirmed lacunar stroke cases. To ensure a fair comparison, we repeated the analysis using SNPs available across all 3 cohorts, and no significant associations of genetically proxied IL‐6R downregulation and small vessel stroke were found (Figure S2 and Table S6). Similar results were obtained for the genetic instrument with 1 missense variant (Table S6).

### Genetically Downregulated IL‐6 Signaling and Imaging and Pathological Arteriolosclerotic cSVD Traits

**Figure 3:**
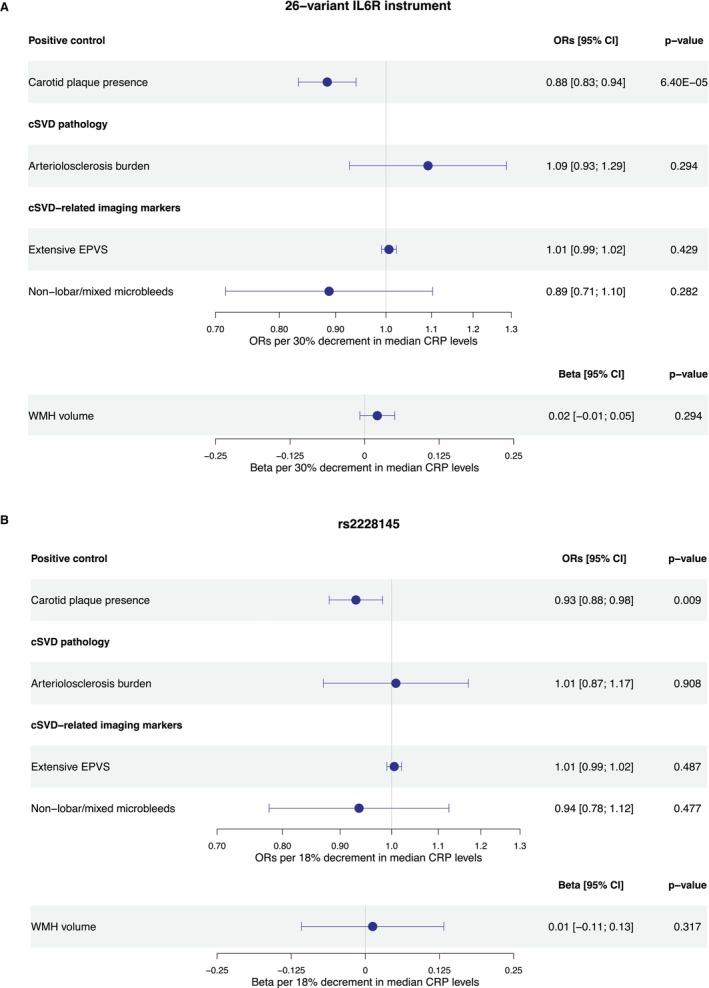
Genetically proxied IL‐6 signaling downregulation and arteriolosclerotic cSVD‐ related imaging and pathology outcomes. Results derived from (**A**) a fixed‐effects inverse‐variance weighted Mendelian randomization analysis for a genetic instrument composed of 26 CRP‐lowering variants in the *IL6R* locus and (**B**) the Wald ratio method for the genetic instrument composed of the single rs2228145 variant. CRP indicates C‐reactive protein; cSVD, cerebral small vessel disease; EPVS, enlarged perivascular spaces; IL‐6, interleukin‐6; *IL6R*, interleukin‐6 receptor; OR, odds ratio; and WMH, white matter hyperintensity.

After correcting for multiple comparisons, genetically proxied downregulation of IL‐6 signaling through either of the 2 instruments in the *IL6R* locus was not associated with arteriolosclerotic cSVD‐related imaging biomarkers (WMH volume, deep/mixed CMBs, extensive basal ganglia EPVS) or arteriolosclerosis burden assessed by autopsy (Figure 3). In contrast, there was a significant association between genetically downregulated IL‐6 signaling and lower odds of a carotid plaque on ultrasound (OR per 30% decrement in CRP levels, 0.88 [95% CI, 0.83–0.94]; *P*=6×10^−5^). These results were consistent across different MR methods (Table S7).

## DISCUSSION

In this integrative analysis of genomic, clinical, imaging, and pathological data, we found no genetic support for IL‐6 signaling as a viable therapeutic target for lowering the burden of arteriolosclerotic cSVD. Specifically, IL‐6 signaling activity, proxied by CRP‐lowering variants near *IL6R*, was not associated with clinical outcomes related to arteriolosclerotic cSVD, including small vessel stroke, imaging‐confirmed lacunar stroke, nonlobar intracerebral hemorrhage, or vascular dementia. Furthermore, there was no significant association of genetically proxied IL‐6 signaling activity with imaging markers of cSVD (WMH, CMB, and EPVS) or arteriolosclerosis pathology burden in the deep perforating arterioles territory of the brain in autopsied individuals.

These findings have important implications for ongoing and planned clinical trials testing anti‐inflammatory treatments for secondary ischemic stroke prevention. Most of the proposed interventions are expected to cause direct or indirect inhibition of the nucleotide‐binding domain leucine‐rich repeat and pyrin domain containing receptor 3 (NLRP3)‐IL‐1β/‐IL‐6 axis, because there is strong evidence for its involvement in atherosclerosis. As confirmed in our current analyses, genetically proxied IL‐6 signaling downregulation was associated with a lower risk of both atherosclerotic stroke and carotid plaque. Inclusion of patients with lacunar stroke or cSVD in such trials has been a matter of controversy. For example, the CONVINCE trial tested colchicine in patients with a recent history of noncardioembolic stroke, including patients with cSVD‐related stroke,^20^ but did not show a significant effect on its primary end point of recurrent vascular events. However, a significant reduction in risk was observed for patients with advanced atherosclerosis, as captured by a >50% carotid stenosis or a history of coronary artery disease. Post hoc analyses will clarify whether the inclusion of patients with lacunar stroke diluted the effect of colchicine in CONVINCE.^20^ Ongoing trials vary in their inclusion criteria; CASPER, for example, includes ischemic strokes of any cause, as long as CRP is elevated, whereas RIISC‐THETIS requires a ≥30% stenosis in a brain‐supplying artery.^21^, ^22^

Our results for lacunar stroke were consistent against a large association with genetically proxied IL‐6 signaling activity and might appear to contradict results from earlier analyses.^29^, ^71^ However, this discrepancy may be related to the inclusion of non‐cSVD‐related lacunar strokes in earlier GWAS of small vessel stroke. Non‐MRI‐defined small vessel strokes are identified by the absence of a larger cortical or subcortical infarct on CT, without evidence of a 50% stenosis in brain‐supplying arteries or a cardioembolic source.^49^ This definition may include smaller infarcts associated with branch atherosclerosis disease affecting small perforating arterioles or other symptomatic nonstenotic atherosclerotic lesions.^73^, ^74^, ^75^ However, after restricting the analysis to MRI‐confirmed lacunar infarcts more likely to be caused by arteriolosclerotic cSVD, we found no significant evidence of an effect of genetically proxied IL‐6 signaling downregulation. The enrichment of the latest small vessel stroke GWAS data set (GIGASTROKE) with MRI‐confirmed lacunar strokes may also explain the negative findings for small vessel stroke in our analysis. Combined with the strong association of genetically proxied IL‐6 signaling with large artery atherosclerotic stroke, these results suggest that the associations observed in earlier data sets may have been driven by a substantial proportion of traditionally defined small vessel strokes caused by atherosclerosis.

This study has limitations. First, an inherent weakness of MR is its assessment of the lifelong effect of genetic perturbation of a drug target, which may not align with the outcomes of a short‐term pharmacological target modulation. Only data from clinical trials testing inhibitors of IL‐6 signaling can directly address this question. Second, we relied on genomic data from primarily European populations, potentially limiting the transferability of our analyses to other ancestries. Third, although we focused on genetic variants near *IL6R* to reduce pleiotropic effects, we cannot fully exclude the potential influence of SNPs impacting neighboring genes, thus introducing potential pleiotropic effects in our analyses. However, the generally consistent results of an analysis focusing on a well‐characterized variant within the exonic sequence of *IL6R* argue against this possibility. Fourth, despite using the largest available data sets for cSVD‐related outcomes, several of these GWAS analyses remain underpowered, meaning that effects of smaller magnitude cannot be entirely ruled out based on our analysis. Finally, there is substantial sample overlap between the GWAS data used to construct the *IL6R* instrument and those for carotid plaque, because both data sets represent meta‐analyses of the UK Biobank and CHARGE consortia, and this overlap might introduce weak‐instrument bias in this analysis.^76^

In conclusion, we found no significant evidence that genetically proxied IL‐6 signaling is associated with clinical, imaging, or pathology‐defined outcomes related to arteriolosclerotic cSVD. Therefore, genetic data do not support the hypothesis that pharmacological therapies targeting IL‐6 signaling would prevent cSVD‐related adverse outcomes.

## Sources of Funding

L.A.T.‐K. receives a Commonwealth PhD scholarship with complementary support from the Nuffield Department of Population Health and the Schlumberger Foundation. J.K.‐T. is supported by a Wellcome Trust Early Career Award (grant number 304532/Z/23/Z). M.K.G. is supported by the Fritz‐Thyssen Foundation (grant reference 10.22.2.024MN), the German Research Foundation (Emmy Noether grant GZ: GE 3461/2‐1, ID 512461526; Munich Cluster for Systems Neurology EXC 2145 SyNergy, ID 390857198), and the Hertie Foundation (Hertie Network of Excellence in Clinical Neuroscience, ID P1230035). H.S.M. and A.S.'s contribution to the study was funded by a British Heart Foundation program grant (RG/F/22/110052). Infrastructural support was provided by the Cambridge British Heart Foundation Center of Research Excellence (RE/18/1/34212) and Cambridge University Hospitals National Institute for Health and Care Research Biomedical Research Centre (BRC‐1215‐20014).

## Disclosures

M.K.G. has received consulting fees from Tourmaline Bio unrelated to this work. The other authors have nothing to disclose.

## Supporting information

Tables S1–S7
Figures S1–S2
