# Supplementary material for: Genetic Downregulation of Interleukin‐6 Signaling and Arteriolosclerotic Cerebral Small Vessel Disease: A Drug Target Mendelian Randomization Analysis
Source: J Am Heart Assoc. 2025 Nov 3;14(21):e041814. doi: 10.1161/JAHA.124.041814 (PMC12684786; doi:10.1161/JAHA.124.041814)

SUPPLEMENTAL MATERIAL

Table S1. STROBE-MR checklist of recommended items to address in reports of Mendelian randomization studies.

Item No.	Section	Checklist item	Page No.	Relevant text from manuscript
1	**TITLE and ABSTRACT**	Indicate Mendelian randomization (MR) as the study's design in the title and/or the abstract if that is a main purpose of the study	1 and 4	Title and Abstract
	**INTRODUCTION**			
2	**Background**	Explain the scientific background and rationale for the reported study. What is the exposure? Is a potential causal relationship between exposure and outcome plausible? Justify why MR is a helpful method to address the study question	6	Introduction Section Paragraph 3 and 4
3	**Objectives**	State specific objectives clearly, including pre-specified causal hypotheses (if any). State that MR is a method that, under specific assumptions, intends to estimate causal effects	6	Introduction Section Paragraph 4
	**METHODS**			
4	**Study design and data sources**	Present key elements of the study design early in the article. Consider including a table listing sources of data for all phases of the study. For each data source contributing to the analysis, describe the following:		
	a)	Setting: Describe the study design and the underlying population, if possible. Describe the setting, locations, and relevant dates, including periods of recruitment, exposure, follow-up, and data collection, when available.	9, 10,11,12,13	Methods (outcomes), Table S1
	b)	Participants: Give the eligibility criteria, and the sources and methods of selection of participants. Report the sample size, and whether any power or sample size calculations were carried out prior to the main analysis	9, 10,11,12,13	Methods (outcomes), Table S1
	c)	Describe measurement, quality control and selection of genetic variants	8, 32	Methods (Genetic instruments) - Figure 1 , Table S2
	d)	For each exposure, outcome, and other relevant variables, describe methods of assessment and diagnostic criteria for diseases	9, 10,11,12,13	Methods (outcomes)
	e)	Provide details of ethics committee approval and participant informed consent, if relevant	14	Methods (ethics)
5	**Assumptions**	Explicitly state the three core IV assumptions for the main analysis (relevance, independence and exclusion restriction) as well assumptions for any additional or sensitivity analysis	8	Methods - paragraph 1
6	**Statistical methods: main analysis**	Describe statistical methods and statistics used	13 and 14	Methods - Statistical analysis
	a)	Describe how quantitative variables were handled in the analyses (i.e., scale, units, model)	13 and 14	Methods - Statistical analysis
	b)	Describe how genetic variants were handled in the analyses and, if applicable, how their weights were selected	13 and 14	Methods - Statistical analysis - Genetic instruments
	c)	Describe the MR estimator (e.g., two-stage least squares, Wald ratio) and related statistics. Detail the included covariates and, in case of two-sample MR, whether the same covariate set was used for adjustment in the two samples	13 and 14	Methods - Statistical analysis
	d)	Explain how missing data were addressed	-	No missing data
	e)	If applicable, indicate how multiple testing was addressed	13 and 14	Methods - Statistical analysis
7	**Assessment of assumptions**	Describe any methods or prior knowledge used to assess the assumptions or justify their validity	7	Introduction, Methods (Genetic instruments)
8	**Sensitivity analyses and additional analyses**	Describe any sensitivity analyses or additional analyses performed (e.g., comparison of effect estimates from different approaches, independent replication, bias analytic techniques, validation of instruments, simulations)	8	Methods (Genetic instruments)
9	**Software and pre-registration**			
	a)	Name statistical software and package(s), including version and settings used	13 and 14	Methods - Statistical analysis
	b)	State whether the study protocol and details were pre-registered (as well as when and where)		Not Needed
	**RESULTS**			
10	**Descriptive data**			
	a)	Report the numbers of individuals at each stage of included studies and reasons for exclusion. Consider use of a flow diagram	NA	NA
	b)	Report summary statistics for phenotypic exposure(s), outcome(s), and other relevant variables (e.g., means, SDs, proportions)	16, 17, 18, 29	Results section1 and table 1, 2
	c)	If the data sources include meta-analyses of previous studies, provide the assessments of heterogeneity across these studies	NA	NA
	d)	For two-sample MR:	NA	NA

		i. Provide justification of the similarity of the genetic variant-exposure associations between the exposure and outcome samples<br>ii. Provide information on the number of individuals who overlap between the exposure and outcome studies		
11	**Main results**			
	a)	Report the associations between genetic variant and exposure, and between genetic variant and outcome, preferably on an interpretable scale	16, 17	results section 2 and 3
	b)	Report MR estimates of the relationship between exposure and outcome, and the measures of uncertainty from the MR analysis, on an interpretable scale, such as odds ratio or relative risk per SD difference	16, 17, 34 , 33	results section 2 and 3, Figure 2 and 3
	c)	If relevant, consider translating estimates of relative risk into absolute risk for a meaningful time period	NA	NA
	d)	Consider plots to visualize results (e.g., forest plot, scatterplot of associations between genetic variants and outcome versus between genetic variants and exposure)	33, 34	Figure 2 and 3
12	**Assessment of assumptions**			
	a)	Report the assessment of the validity of the assumptions		
	b)	Report any additional statistics (e.g., assessments of heterogeneity across genetic variants, such as I^2, Q statistic or E-value)		Table S4 and Table S5 (Q, p value of MR Egger)
13	**Sensitivity analyses and additional analyses**			
	a)	Report any sensitivity analyses to assess the robustness of the main results to violations of the assumptions		Table S4 and Table S5 (MR-Egger, weighted median)
	b)	Report results from other sensitivity analyses or additional analyses		Table S4 and 5 (weighted mode)
	c)	Report any assessment of direction of causal relationship (e.g., bidirectional MR)	16, 17, 34 , 33	Results, Figure 2 and 3
	d)	When relevant, report and compare with estimates from non-MR analyses	11, 12	Discussion
	e)	Consider additional plots to visualize results (e.g., leave-one-out analyses)		
	**DISCUSSION**			
14	**Key results**	Summarize key results with reference to study objectives	18	Discussion paragraph 1
15	**Limitations**	Discuss limitations of the study, taking into account the validity of the IV assumptions, other sources of potential bias, and imprecision. Discuss both direction and magnitude of any potential bias and any efforts to address them	19	Discussion paragraph 4
16	**Interpretation**			
	a)	Meaning: Give a cautious overall interpretation of results in the context of their limitations and in comparison, with other studies	19	Discussion paragraph 2 and 3
	b)	Mechanism: Discuss underlying biological mechanisms that could drive a potential causal relationship between the investigated exposure and the outcome, and whether the gene-environment equivalence assumption is reasonable. Use causal language carefully, clarifying that IV estimates may provide causal effects only under certain assumptions	19	Discussion paragraph 2 and 3
	c)	Clinical relevance: Discuss whether the results have clinical or public policy relevance, and to what extent they inform effect sizes of possible interventions	19, 20	Discussion paragraph 2 and 3 and 5
17	**Generalizability**	Discuss the generalizability of the study results (a) to other populations, (b) across other exposure periods/timings, and (c) across other levels of exposure	19, 20	Discussion paragraph 2 , 3 and 4
	**OTHER INFORMATION**			
18	**Funding**	Describe sources of funding and the role of funders in the present study and, if applicable, sources of funding for the databases and original study or studies on which the present study is based	20	Source of funding section
19	**Data and data sharing**	Provide the data used to perform all analyses or report where and how the data can be accessed, and reference these sources in the article. Provide the statistical code needed to reproduce the results in the article, or report whether the code is publicly accessible and if so, where	15	Data availability
20	**Conflicts of Interest**	All authors should declare all potential conflicts of interest	21	Disclosures

Table S2. Data sources for GWAS studies included in this analysis.

	Exposure/Outcome	Source Study. Year [reference number in Manuscript]	Ancestry description	Imputation panel	Adjustments	Phenotype scale	Cases / Controls/ Sample size
				Exposure			
1	**IL6 receptor-mediated signaling**	Georgakis et al. 2022 [31]	European (100%)	HRC panel, UK10K panel, 1000 Genomes phase 3, HapMap,1000 Genomes Phase I	Age, sex and population substructure/ statins, sex, Townsend Deprivation Index at baseline, the top 20 PCs of the genotype matrix, age at baseline, BMI at baseline, age difference between baseline and follow-up, BMI difference between baseline and follow-up, and baseline age, age difference, and BMI difference by sex interactions	continuous log transformed	575,531 participants
				Outcome 1: Clinical manifestations attributed to arteriolosclerotic cSVD			
3	**Small vessel stroke MEGASTROKE European**	Malik et al.2018 [51]	European (100%)	1000 Genomes phase 1	At least sex and age and specific covariates when needed	binary trait	5,386 cases and 343,560 controls
4	**Small vessel stroke GIGASTROKE Trans-ancestry**	Mishra et al. 2022[50]	Trans-ancestry (67% European, 25% East Asian, 4% African, 3% South Asian, 1% Hispanic)	HRC / 1000 Genomes phase 1 or phase 3 / TOPMed / HapMap / biobank-specific reference panels	Age, sex, principal components of population stratification, and study-specific covariates when needed	binary trait	13,620 cases and 1,503,898 controls
5	**Small vessel stroke GIGASTROKE European**	Mishra et al. 2022[50]	European (100%)	HRC / 1000 Genomes phase 1 or phase 3 / TOPMed / HapMap / biobank-specific reference panels	Age, sex, principal components of population stratification, and study-specific covariates when needed	binary trait	6,811 cases and 1,234,808 controls
6	**MRI-confirmed Lacunar stroke**	Kilarski et al. 2015[53] and UK DNA Lacunar Stroke 1 and 2 studies	European (100%)	TOPMed	Sex, principal components, cryptic relatedness, Firth correction	binary trait	2,612 + 686 new cases (3,298) and 19,948 controls
7	**Non-lobar Intracerebral hemorrhage**	Woo et al. 2014 [54]	European (100%)	1000 Genomes phase 1	Age, sex, and first 4 principal components	binary trait	881 cases and 1,481 controls
8	**Vascular dementia**	Mega Vascular Cognitive Impairment and Dementia (MEGAVCID) consortium. 2024 [56]	Predominantly European (98% European, 1% African, <1% Asian, <1% Hispanics)	HRC / 1000 Genomes/ UK10K/ TOPMed	Age, sex, sites, and population structure	binary trait	8,702 cases and 753,695 controls
				Outcome 2: Imaging biomarkers of arteriolosclerotic cSVD			
9	**White matter hyperintensity**	Sargurupremraj et al. 2020[57]	European (100%)	UK10K / HRC / 1000 Genomes phase 1	Age, sex, principal components and intracranial volume	inverse normal transformed	48,454 participants
10	**Enlarged perivascular spaces**	Duperron et al. 2023[58]	Predominantly European (97% European, < 1% Hispanic, <1% East Asian, 1,8% African-American)	1000 Genomes phase 1/ HRC	Age, sex and intracranial volume/brain parenchymal fraction, principal components of population stratification, and study site	binary trait	9,189 cases and 30,811 controls
11	**Cerebral microbleeds**	Knol et al. 2020[60]	Predominantly European (97% European, 1.6 % African American, <1% Malay, <1% Chinese)	HRC / 1000 Genomes	Age, sex, and principal components of ancestry and family relations	binary trait	1,293 cases and 24,569 controls
				Outcome 3: Pathology burden of arteriolosclerosis			

12	**Arteriolosclerosis burden**	Shade et al. 2024[61]	European (100%)	TOPMed	Age at death, sex, genotyping cohort and top ten genetic PCs	ordinal trait	6,668 autopsied participants with 1,645 having no arteriolosclerosis, 2,122 with mild arteriolosclerosis, 2,067 with moderate arteriolosclerosis, 834 with severe arteriolosclerosis
			Positive control: Atherosclerosis-related traits				
13	**Large artery atherosclerotic stroke - GIGASTROKE Trans-ancestry**	Mishra et al. 2022[50]	Trans-ancestry (67% European, 25% East Asian, 4% African, 3% South Asian, 1% Hispanic)	HRC / 1000 Genomes phase 1 or phase 3 reference panels / TOPMed / HapMap / biobank-specific reference panels	Age, sex, principal components of population stratification, and study-specific covariates when needed	binary trait	9,219 cases and 1,503,898 controls
14	**Large artery atherosclerotic stroke - GIGASTROKE European**	Mishra et al. 2022[50]	European (100%)	HRC / 1000 Genomes phase 1 or phase 3 reference panels / TOPMed / HapMap / biobank-specific reference panels	Age, sex, principal components of population stratification, and study-specific covariates when needed	binary trait	6,399 cases and 1,234,808 controls
15	**Presence of carotid atherosclerotic plaques**	Omarov et al. 2024[64]	European (100%)	1000 Genomes / HRC / UK10K	Sex, age, the first 10 principal components and the genotyping chip	binary trait	29,790 cases and 36,847 controls

Table S3. List of genetic instruments.

SNP	chromosome	bp_hg19	effect allele	other allele	beta	stderr	Pval	F-statistic
rs112203594	1	154553430	a	c	0.0396	0.0071	2.09E-08	31.108
rs11264224	1	154568086	a	c	0.0418	0.0028	1.6E-49	222.862
rs113580743	1	154420333	a	g	0.055	0.0055	1.09E-23	100.000
rs116141616	1	154416069	a	g	0.0387	0.0069	1.76E-08	31.457
rs12059682	1	154579585	c	t	0.0474	0.0025	2.11E-77	359.482
rs12083537	1	154381103	a	g	0.0679	0.0026	3.03E-156	682.013
rs12406117	1	154740879	a	g	0.0124	0.0021	3.83E-09	34.866
rs12735458	1	154361406	a	g	0.0842	0.0092	4.53E-20	83.762
rs144029367	1	154455249	c	t	0.0498	0.008	5.43E-10	38.751
rs145262901	1	154394484	g	a	0.061	0.0102	2.38E-09	35.765
rs145909430	1	154391504	t	c	0.1001	0.0082	2.86E-34	149.019
rs16836054	1	154462195	a	g	0.0516	0.0028	1.51E-75	339.612
rs183641528	1	154499328	g	a	0.0851	0.008	1.62E-26	113.156
rs2228145	1	154426970	a	c	0.0947	0.0021	3E-307	2033.580
rs34693607	1	154661369	c	g	0.0328	0.0026	3.83E-36	159.148
rs3738028	1	154698817	a	c	0.0137	0.0023	1.19E-09	35.480
rs3766925	1	154564712	t	a	0.0148	0.0025	2.69E-09	35.046
rs41269913	1	154461480	c	t	0.0424	0.0058	2.28E-13	53.441
rs56100876	1	154496473	g	a	0.117	0.0086	3.27E-42	185.087
rs61806853	1	154154587	t	c	0.0437	0.005	1.23E-18	76.388
rs6698385	1	154652572	g	a	0.035	0.0026	3.74E-41	181.213
rs73026617	1	154369981	t	c	0.0467	0.0034	1.69E-42	188.658
rs7525477	1	154394297	a	g	0.0296	0.0023	1.35E-38	165.626
rs76289529	1	154516404	c	t	0.0519	0.006	4.78E-18	74.823
rs77994623	1	154505106	t	c	0.046	0.0029	1.5E-58	251.605
rs78035035	1	154273429	a	c	0.0457	0.0081	1.43E-08	31.832

Table S4. Mendelian randomization power calculations for clinical outcomes.

Outcome	Cases	Controls	0 < OR <=1	OR > 1
Large artery stroke	9219	1503898	0.88	1.13
Small Vessel stroke	13620	1503898	0.9	1.1
MRI-confirmed lacunar stroke	3298	19948	0.78	1.26
Primary spontaneous intracerebral hemorrhage	881	1481	0.61	1.63
Vascular dementia	8702	753695	0.87	1.13

R2	0.00987
alpha (Type-I error rate)	0.05
1 - beta (required power)	0.80

Table S5. Mendelian randomization results for clinical outcomes.

Results for downregulated IL-6 signaling proxied by the 26-variant genetic instruments																					
MR RESULTS												*F-STATISTICS*		*Heterogeneity method*				*Pleiotropy results*			
	**Outcomes**	**Method**	**Number of SNPS**	**b (1-unit increment in lnCRP)**	**beta (30% decrement in CRP)**	**OR (30% decrement in CRP)**	**LCI**	**UCI**	**pval**	**se (1-unit increment in lnCRP)**	**se (30% decrement in CRP)**	**Statistic**	**Estimate**	**statistic**	**Q**	**Q_df**	**Q_pval**	**statistic**	**egger.intercept**	**pleiotropy.pval**	**pleiotropy.se**
1	Large artery stroke -GIGASTROKE - TRANSANCESTRY	IVW - FE	15	0.652	-0.232	0.79	0.72	0.87	<0.001	0.141	0.050	Mean F-statistic	324.372	IVW	5.639	14	0.975	Egger intercept	0.005	0.700	0.013
		MR Egger	15	0.553	-0.197	0.82	0.67	1.00	0.077	0.288	0.103			MR Egger	5.484	13	0.963				
		Weighted median	15	0.664	-0.237	0.79	0.69	0.90	<0.001	0.184	0.065										
		Weighted mode	15	0.681	-0.243	0.78	0.69	0.90	0.003	0.192	0.068										
2	Non-lobar ICH	IVW - FE	8	-0.107	0.038	1.04	0.72	1.50	0.837	0.521	0.186	Mean F-statistic	406.601	IVW	2.626	7	0.917	Egger intercept	0.083	0.254	0.065
		MR Egger	8	-1.356	0.484	1.62	0.74	3.54	0.271	1.118	0.399			MR Egger	1.034	6	0.984				
		Weighted median	8	-0.308	0.110	1.12	0.73	1.70	0.606	0.599	0.213										
		Weighted mode	8	-0.348	0.124	1.13	0.73	1.76	0.597	0.628	0.224										
3	Small Vessel Stroke - GIGASTROKE - TRANSANCESTRY	IVW - FE	14	-0.066	0.023	1.02	0.95	1.10	0.536	0.106	0.038	Mean F-statistic	342.085	IVW	19.557	13	0.107	Egger intercept	-0.003	0.839	0.012
		MR Egger	14	-0.017	0.006	1.01	0.83	1.22	0.950	0.270	0.096			MR Egger	19.487	12	0.077				
		Weighted median	14	-0.013	0.004	1.00	0.90	1.12	0.935	0.153	0.054										
		Weighted mode	14	-0.071	0.025	1.03	0.92	1.15	0.659	0.158	0.056										
4	Lacunar Stroke - MRI Defined	IVW - FE	21	0.148	-0.053	0.95	0.81	1.11	0.505	0.222	0.079	Mean F-statistic	250.170	IVW	17.765	20	0.603	Egger intercept	-0.011	0.693	0.027
		MR Egger	21	0.337	-0.120	0.89	0.62	1.28	0.526	0.522	0.186			MR Egger	17.604	19	0.549				
		Weighted median	21	0.144	-0.051	0.95	0.77	1.18	0.640	0.309	0.110										
		Weighted mode	21	0.194	-0.069	0.93	0.75	1.16	0.544	0.315	0.112										
5	Vascular Dementia	IVW - FE	17	-0.238	0.085	1.09	0.95	1.25	0.221	0.194	0.069	Mean F-statistic	293.756	IVW	33.314	16	0.007	Egger intercept	0.009	0.757	0.027
		MR Egger	17	-0.392	0.140	1.15	0.77	1.71	0.501	0.569	0.203			MR Egger	33.095	15	0.005				
		Weighted median	17	-0.173	0.062	1.06	0.88	1.29	0.522	0.271	0.097										
		Weighted mode	17	-0.076	0.027	1.03	0.85	1.25	0.788	0.277	0.099										
6	Large artery stroke -GIGASTROKE - EUROPEAN	IVW - FE	15	0.751	-0.268	0.77	0.68	0.87	<0.001	0.179	0.064	Mean F-statistic	324.372	IVW	7.021	14	0.934	Egger intercept	0.010	0.551	0.016
		MR Egger	15	0.550	-0.196	0.82	0.63	1.07	0.166	0.374	0.133			MR Egger	6.647	13	0.919				
		Weighted median	15	0.667	-0.238	0.79	0.67	0.93	0.005	0.237	0.085										
		Weighted mode	15	0.725	-0.259	0.77	0.63	0.94	0.021	0.280	0.100										

7	Small Vessel stroke -GIGASTROKE - EUROPEAN	IVW - FE	14	0.197	-0.070	0.93	0.82	1.06	0.282	0.183	0.065	Mean F-statistic	202.286	IVW	7.859	13	0.853	Egger intercept	-0.010	0.558	0.016
		MR Egger	14	0.422	-0.150	0.86	0.64	1.15	0.330	0.416	0.148			MR Egger	7.496	12	0.823				
		Weighted median	14	0.178	-0.063	0.94	0.80	1.11	0.447	0.234	0.083										
		Weighted mode	14	0.236	-0.084	0.92	0.75	1.12	0.424	0.286	0.102										

Results for single variant instrument (rs2228145)

	MR RESULTS											***F-STATISTICS***	
	**Outcomes**	**Method**	**Number of SNPS**	**b**	**beta (18% decrement)**	**OR (18% decrement)**	**LCI**	**UCI**	**pval**	**se**	**se(18% decrement)**	**Statistic**	**Estimate**
1	Large artery stroke - GIGASTROKE - TRANSANCESTRY	Wald ratio	1	0.692	-0.139	0.87	0.78	0.97	0.011	0.273	0.055	Mean F-statistic	2033.580
2	Non-lobar ICH	Wald ratio	1	-0.528	0.106	1.11	0.86	1.44	0.424	0.661	0.133		
3	Small Vessel Stroke - GIGASTROKE - TRANSANCESTRY	Wald ratio	1	-0.335	0.067	1.07	0.99	1.15	0.072	0.186	0.037		
4	Lacunar Stroke - MRI Defined	Wald ratio	1	0.129	-0.026	0.97	0.85	1.12	0.718	0.356	0.072		
5	Vascular Dementia	Wald ratio	1	-0.202	0.041	1.04	0.92	1.17	0.507	0.304	0.061		
6	Large artery stroke -GIGASTROKE - EUROPEAN	Wald ratio	1	1.045	-0.210	0.81	0.69	0.96	0.012	0.417	0.084		
7	Small Vessel stroke -GIGASTROKE - EUROPEAN	Wald ratio	-	-	-	-	-	-	-	-	-		

IVW: inverse-variance weighted Mendelian randomization , FE : fixed effects. MRE: multiplicative random effects, ICH: intracerebral hemorrhage, OR: odd ratios, LCI: lower confidence interval , UCI: upper confidence interval

Table S6. Mendelian randomization results across evolving GWAS datasets.

Results for downregulated IL-6 signaling proxied by the 26-variant genetic instruments

MR RESULTS											*F-STATISTICS*		*Heterogeneity method*				*Pleiotropy results*			
**Outcomes**	**Method**	**Number of SNPS**	**b (1-unit increment in lnCRP)**	**beta (30% decrement in CRP)**	**OR (30% decrement in CRP)**	**LCI**	**UCI**	**pval**	**se (1-unit increment in lnCRP)**	**se (30% decrement in CRP)**	**Statistic**	**Estimate**	**statistic**	**Q**	**Q_df**	**Q_pval**	**statistic**	**egger.intercept**	**pleiotropy.pval**	**pleiotropy.se**
Small Vessel Stroke MEGASTROKE - EUR	IVW - FE	26	0.448	-0.160	0.85	0.77	0.95	0.003	0.150	0.054	Mean F-statistic	218.992	IVW	23.800	25	0.531	Egger intercept	-0.009	0.518	0.014
	MR Egger	26	0.616	-0.220	0.80	0.65	0.99	0.048	0.296	0.106			MR Egger	23.370	24	0.498				
	Weighted median	26	0.487	-0.174	0.84	0.73	0.97	0.022	0.212	0.075										
	Weighted mode	26	0.484	-0.173	0.84	0.73	0.97	0.021	0.197	0.070										
Lacunar Stroke - MRI Defined – EUR	IVW - FE	21	0.148	-0.053	0.95	0.81	1.11	0.505	0.222	0.079	Mean F-statistic	250.170	IVW	17.765	20	0.603	Egger intercept	-0.011	0.693	0.027
	MR Egger	21	0.337	-0.120	0.89	0.62	1.28	0.526	0.522	0.186			MR Egger	17.604	19	0.549				
	Weighted median	21	0.144	-0.051	0.95	0.77	1.17	0.635	0.304	0.108										
	Weighted mode	21	0.194	-0.069	0.93	0.75	1.16	0.542	0.314	0.112										
Small Vessel Stroke GIGASTROKE - EUR	IVW - FE	14	0.197	-0.070	0.93	0.82	1.06	0.282	0.183	0.065	Mean F-statistic	202.286	IVW	7.859	13	0.853	Egger intercept	-0.010	0.558	0.016
	MR Egger	14	0.422	-0.150	0.86	0.64	1.15	0.330	0.416	0.148			MR Egger	7.496	12	0.823				
	Weighted median	14	0.178	-0.063	0.94	0.80	1.11	0.447	0.234	0.083										
	Weighted mode	14	0.236	-0.084	0.92	0.75	1.12	0.424	0.286	0.102										

Sensitivity analysis results with a common subset of SNPs

MR RESULTS											*F-STATISTICS*		*Heterogeneity method*				*Pleiotropy results*			
**Outcomes**	**Method**	**Number of SNPS**	**b (1-unit increment in lnCRP)**	**beta (30% decrement in CRP)**	**OR (30% decrement in CRP)**	**LCI**	**UCI**	**pval**	**se (1-unit increment in lnCRP)**	**se (30% decrement in CRP)**	**Statistic**	**Estimate**	**statistic**	**Q**	**Q_df**	**Q_pval**	**statistic**	**egger.intercept**	**pleiotropy.pval**	**pleiotropy.se**
Small Vessel Stroke MEGASTROKE - EUR	IVW - FE	12	0.492	-0.175	0.84	0.72	0.97	0.020	0.212	0.076	Mean F-statistic	230.138	IVW	4.976	11	0.932	Egger intercept	-0.016	0.578	0.028
	MR Egger	12	0.844	-0.301	0.74	0.47	1.16	0.222	0.648	0.231			MR Egger	4.645	10	0.914				
	Weighted median	12	0.558	-0.199	0.82	0.67	1.00	0.045	0.279	0.099										
	Weighted mode	12	0.521	-0.186	0.83	0.65	1.06	0.157	0.343	0.122										
Lacunar Stroke - MRI Defined – EUR	IVW - FE	12	0.339	-0.121	0.89	0.72	1.10	0.268	0.306	0.109	Mean F-statistic	230.138	IVW	7.883	11	0.724	Egger intercept	-0.030	0.450	0.038
	MR Egger	12	1.000	-0.357	0.70	0.37	1.31	0.290	0.896	0.319			MR Egger	7.266	10	0.700				
	Weighted median	12	0.355	-0.126	0.88	0.66	1.18	0.390	0.413	0.147										
	Weighted mode	12	0.648	-0.231	0.79	0.53	1.18	0.276	0.564	0.201										
Small Vessel Stroke	IVW - FE	12	0.209	-0.075	0.93	0.82	1.06	0.259	0.186	0.066	Mean F-statistic	230.138	IVW	7.490	11	0.758	Egger intercept	-0.012	0.647	0.025

GIGASTROKE - EUR	MR Egger	12	0.464	-0.165	0.85	0.57	1.26	0.434	0.569	0.203			MR Egger	7.266	10	0.700
	Weighted median	12	0.197	-0.070	0.93	0.79	1.11	0.421	0.245	0.087						
	Weighted mode	12	0.188	-0.067	0.94	0.75	1.17	0.564	0.315	0.112						

Results for single variant instrument (rs2228145)												
MR RESULTS											***F-STATISTICS***	
**Outcomes**	**Method**	**Number of SNPS**	**b**	**beta (18% decrement)**	**OR (18% decrement)**	**LCI**	**UCI**	**pval**	**se**	**se(18% decrement)**	**Statistic**	**Estimate**
Small Vessel Stroke MEGASTROKE - EUROPEAN	Wald ratio	1	0.484	-0.097	0.91	0.82	1.00	0.048	0.245	0.049	Mean F-statistic	2033.580
Lacunar Stroke - MRI Defined	Wald ratio	1	0.129	-0.026	0.97	0.85	1.12	0.718	0.356	0.072		
Small Vessel Stroke GIGASTROKE - EUROPEAN	Wald ratio	-	-	-	-	-	-	-	-	-		

IVW: inverse-variance weighted Mendelian randomization , FE : fixed effects. MRE: multiplicative random effects, ICH: intracerebral hemorrhage, OR: odd ratios, LCI: lower confidence interval , UCI: upper confidence interval

Table S7. Mendelian randomization results for imaging and pathological arteriolosclerotic cSVD.

Results for downregulated IL-6 signaling proxied by the 26-variant genetic instruments

	MR RESULTS											*F-STATISTICS*		*Heterogeneity method*				*Pleiotropy results*			
	**Outcomes**	**Method**	**Number of SNPS**	**b (1-unit increment in lnCRP)**	**beta (30% decrement in CRP)**	**OR (30% decrement in CRP)**	**LCI**	**UCI**	**pval**	**se (1-unit increment in lnCRP)**	**se (30% decrement in CRP)**	**Statistic**	**Estimate**	**statistic**	**Q**	**Q_df**	**Q_pval**	**statistic**	**egger.intercept**	**pleiotropy.pval**	**pleiotropy.se**
1	Extensive EPVS	IVW - FE	26	-0.017	0.006	1.006	0.991	1.022	0.429	0.022	0.008	Mean F-statistic	218.992	IVW	26.216	25	0.396	Egger intercept	-0.001	0.708	0.002
		MR Egger	26	-0.002	0.001	1.001	0.969	1.033	0.957	0.046	0.016			MR Egger	26.060	24	0.350				
		Weighted median	26	-0.024	0.008	1.009	0.986	1.032	0.464	0.033	0.012										
		Weighted mode	26	-0.012	0.004	1.004	0.981	1.029	0.728	0.034	0.012										
2	Mixed or strictly deep CMB	IVW - FE	13	0.334	-0.119	0.888	0.715	1.103	0.282	0.310	0.111	Mean F-statistic	360.707	IVW	9.178	12	0.688	Egger intercept	-0.002	0.943	0.028
		MR Egger	13	0.371	-0.132	0.876	0.579	1.325	0.544	0.592	0.211			MR Egger	9.172	11	0.606				
		Weighted median	13	0.453	-0.161	0.851	0.651	1.112	0.238	0.383	0.137										
		Weighted mode	13	0.459	-0.164	0.849	0.655	1.101	0.240	0.371	0.132										
3	Carotid plaques	IVW - FE	26	0.344	-0.123	0.884	0.833	0.939	<0.001	0.086	0.031	Mean F-statistic	218.992	IVW	31.627	25	0.169	Egger intercept	0.007	0.480	0.010
		MR Egger	26	0.224	-0.080	0.923	0.806	1.058	0.260	0.194	0.069			MR Egger	30.962	24	0.155				
		Weighted median	26	0.355	-0.126	0.881	0.811	0.958	0.003	0.120	0.043										
		Weighted mode	26	0.340	-0.121	0.886	0.819	0.959	0.006	0.113	0.040										
4	WMH volume	IVW - FE	10	-0.060	0.021	--	--	--	0.154	0.042	0.015	Mean F-statistic	432.894	IVW	3.471	9	0.943	Egger intercept	0.003	0.482	0.005
		MR Egger	10	-0.115	0.041	--	--	--	0.218	0.086	0.031			MR Egger	2.927	8	0.939				
		Weighted median	10	-0.061	0.022	--	--	--	0.235	0.051	0.018										
		Weighted mode	10	-0.055	0.020	--	--		0.361	0.057	0.021										
5	Arteriolosclerosis burden	IVW - FE	18	-0.247	0.088	1.092	0.927	1.287	0.294	0.235	0.084	Mean F-statistic	279.183	IVW	13.462	17	0.705	Egger intercept	-0.023	0.310	0.022
		MR Egger	18	0.181	-0.064	0.938	0.675	1.303	0.706	0.470	0.168			MR Egger	12.362	16	0.719				
		Weighted median	18	-0.042	0.015	1.015	0.811	1.271	0.897	0.322	0.115										
		Weighted mode	18	0.055	-0.019	0.981	0.774	1.243	0.874	0.338	0.121										

Results for single variant instrument (rs2228145)

	MR RESULTS											*F-STATISTICS*	
	**Outcomes**	**Method**	**Number of SNPS**	**b**	**beta (18% decrement)**	**OR (18% decrement)**	**LCI**	**UCI**	**pval**	**se**	**se(18% decrement)**	**Statistic**	**Estimate**
1	Extensive EPVS	Wald ratio	1	-0.026	0.005	1.005	0.990	1.020	0.487	0.038	0.008	Mean F-statistic	2033.580
2	Mixed or strictly deep CMB	Wald ratio	1	0.332	-0.067	0.936	0.778	1.124	0.477	0.467	0.094		

3	Carotid plaques	Wald ratio	1	0.363	-0.073	0.930	0.880	0.982	0.009	0.138	0.028	
4	WMH volume	Wald ratio	1	-0.061	0.012	--	--	--	0.317	0.061	0.012	
5	Arteriolosclerosis burden	Wald ratio	1	-0.043	0.009	1.009	0.870	1.170	0.908	0.376	0.076	

IVW: inverse-variance weighted Mendelian randomization , FE : fixed effects. ICH: intracerebral hemorrhage, OR: odd ratios, LCI: lower confidence interval , UCI: upper confidence interval, CMB: cerebral microbleeds, WMH: white matter hyperintensity , EPVS: extensive Perivascular spaces

Figure S1. Leave-one-out sensitivity analysis results for clinical outcomes from Mendelian Randomization using a 26-variant instrument.

Each point represents the Inverse-Variance Weighted fixed-effects (IVW-FE) estimate obtained by excluding one genetic variant at a time. Horizontal lines denote 95% confidence intervals (CIs). Each plot, corresponding to a specific clinical outcome, contains the number of genetic variants available in the respective summary statistics.

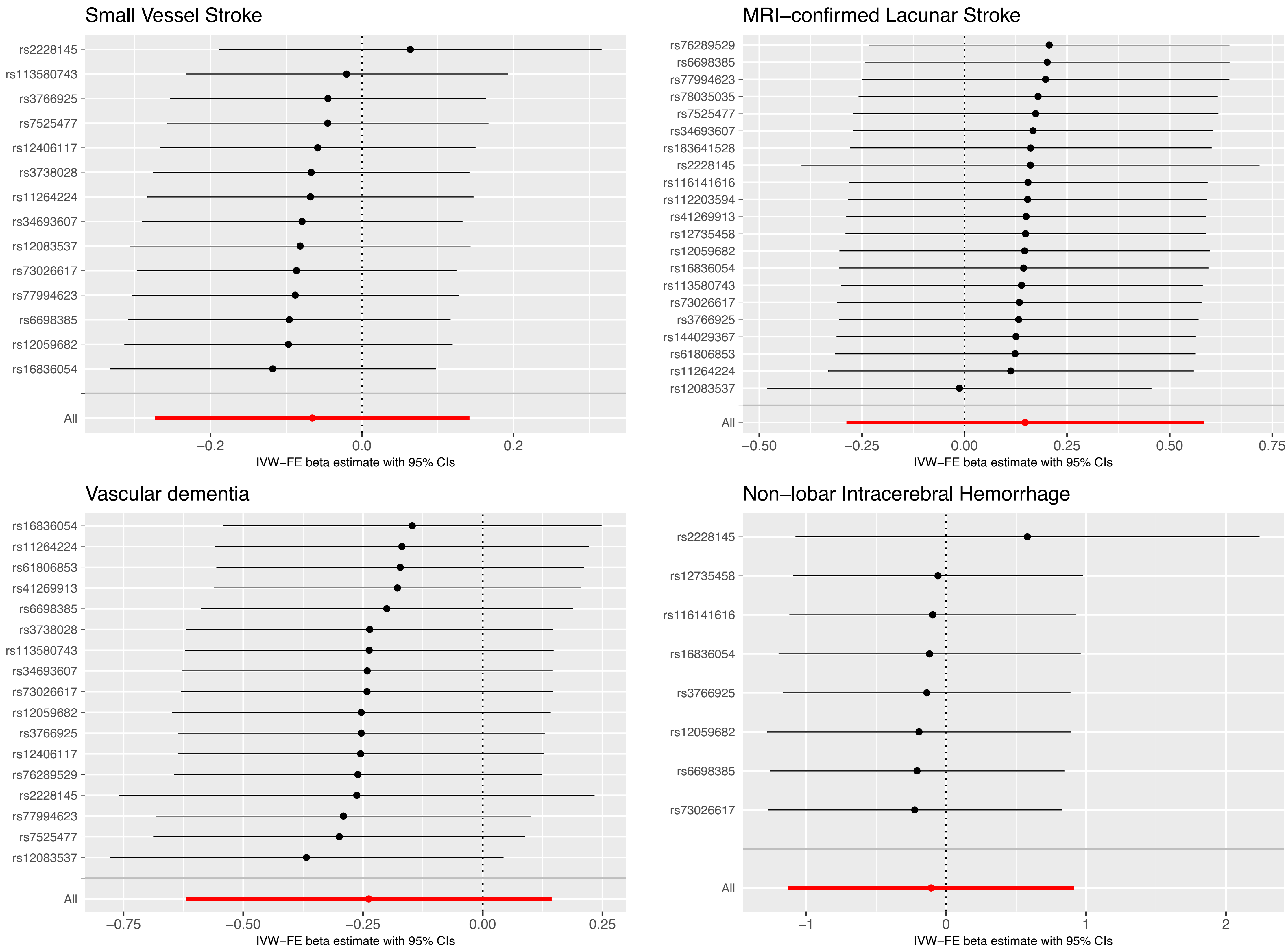

Figure S2. Genetically downregulated IL-6 signaling and small vessel stroke across the datasets.

Results derived from fixed-effects inverse-variance weighted Mendelian randomization analysis for a genetic instrument composed of 12 common CRP-lowering variants in the IL6R locus.

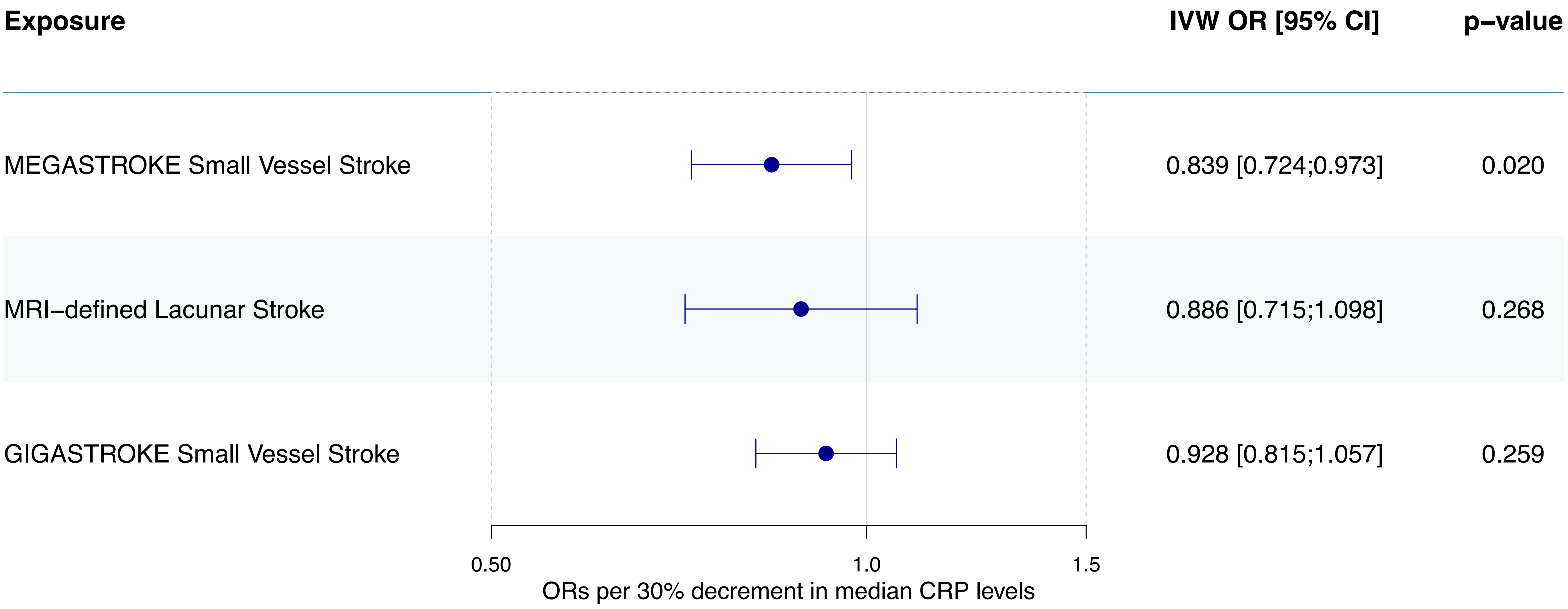

Supplement: Supplementary file 1 — Tables S1–S7 Figures S1–S2 [file JAH3-14-e041814-s001.pdf]
